# Discovery of chemerin as the new chemoattractant of human mesenchymal stem cells

**DOI:** 10.1186/s13578-021-00631-3

**Published:** 2021-07-01

**Authors:** Irene Kim, Hyomin Park, Injoo Hwang, Dodam Moon, Hyunji Yun, Eun Ju Lee, Hyo-Soo Kim

**Affiliations:** 1 Molecular Medicine & Biopharmaceutical Sciences, Graduate School of Convergence Science and Technology, and College of Medicine, Seoul National University, Seoul National University Hospital, 101 DeaHak-ro, JongRo-gu, Seoul, 03080 Republic of Korea; 2 Program in Stem Cell Biology, Seoul National University College of Medicine, Seoul, Republic of Korea; 3 Biomedical Research Institute, Seoul National University Hospital, 101 DeaHak-ro, JongRo-gu, Seoul, 03080 Republic of Korea; 4 Department of Internal Medicine, Seoul National University College of Medicine, Seoul, Republic of Korea

**Keywords:** Active chemerin, Chemoattractant, Intravascularly-delivery, Homing of stem cells

## Abstract

**Background:**

The homing capacity of human mesenchymal stem cells (hMSCs) to the injured sites enables systemic administration of hMSCs in clinical practice. In reality, only a small proportion of MSCs are detected in the target tissue, which is a major bottleneck for MSC-based therapies. We still don’t know the mechanism how MSCs are chemo-attracted to certain target organ and engrafted through trans-endothelial migration. In this study, we aimed to determine the mechanism how the circulating hMSCs home to the injured liver.

**Methods and results:**

When we compare the cytokine array between normal and injured mouse liver at 1-day thioacetamide (TAA)-treatment, we found that chemerin, CXCL2, and CXCL10 were higher in the injured liver than normal one. Among three, only chemerin was the chemoattractant of hMSCs in 2D- and 3D-migration assay. Analysis of the signal transduction pathways in hMSCs showed that chemerin activated the phosphorylation of JNK1/2, ERK1/2 and p38, and finally upregulated CD44, ITGA4, and MMP-2 that are involved in the transendothelial migration and extravasation of MSCs. Upstream transcription regulators of *CD44*, *ITGA4*, and *MMP-2* after chemerin treatment were MZF1, GATA3, STAT3, and STAT5A. To develop chemerin as a chemoattractant tool, we cloned gene encoding the active chemerin under the CMV promoter (CMV-aChemerin). We analyzed the migration of hMSCs in the 3D model for space of the Disse, which mimics transmigration of hMSCs in the liver. CMV-aChemerin-transfected hepatocytes were more effective to attract hMSC than control hepatocytes, leading to the enhanced transendothelial migration and homing of hMSCs to liver. The homing efficiency of the intravascularly-delivered hMSCs to liver was evaluated after systemic introduction of the CMV-aChemerin plasmid packed in liposome-vitamin A conjugates which target liver. CMV-aChemerin plasmid targeting liver significantly enhanced homing efficiency of hMSCs to liver compared with control plasmid vector.

**Conclusions:**

Chemerin is the newly found chemoattractant of hMSCs and may be a useful tool to manipulate the homing of the intravascularly-administered hMSC to the specific target organ.

**Supplementary Information:**

The online version contains supplementary material available at 10.1186/s13578-021-00631-3.

## Background

The homing capacity of mesenchymal stem cells (MSCs), which allows the cells to navigate to injured sites, enables systemic administration of MSCs in clinical practice. Homing includes transendothelial migration, which is guided by certain chemokines from the injured site. However, only a small proportion of MSCs is detected in the target tissue, which has been a major bottleneck for MSC-based therapies [1].

The mechanisms underlying leukocyte and lymphocyte chemotactic responses and their attachment and penetration through endothelial cells (ECs) are well-understood; however, we don’t know well the mechanism how MSCs are chemoattracted and migrate through endothelial barrier toward the final destination [2] or the injured liver in our study.

To improve the low efficacy of homing to the target sites via transendothelial migration, chemokines released from the injured tissue are reliable candidates of chemoattractants. Indeed, local inflammation induces homing of hMSCs as well as engraftment in mice [3]. Stromal cell derived factor (SDF)-1 from endothelial cells and its interaction with CXCR4, which is expressed in MSCs, is critical for this step [4, 5]. Therefore, studies have focused on raising homing efficiency of MSCs using CXCR4/SDF-1α axis [4–6]. Studies have shown that the CXCR4/SDF-1 axis is crucially involved in hematopoietic stem cell (HSC) homing in bone marrow niches [7]. However, the expression of CXCR4 on the surface of MSCs is controversial. Some groups have reported that CXCR4 is important for MSC migration to the bone marrow, although its expression levels are low [8]. Other groups have reported that MSCs do not express CXCR4 [9]. Another study showed that MSCs express various other receptors and migrate toward a wide spectrum of signals [10–12]. Using these factors, as well as the CXCR4/SDF-1 axis, various strategies have been applied to improve this step [1, 13]. However, homing of MSCs to target tissue after systemic administration is still inefficient.

Chemerin is a chemotactic protein that is mostly synthesized and secreted by the liver, adipocytes, and lung [14]. It is secreted as an inactive precursor named pro-chemerin and is activated by different proteases depending on the injured site [14]. The last 6 to 7 amino acids at the C terminus of pro-chemerin are cleaved to obtain biologically active chemerin [15, 16]. Active chemerin mainly interacts with the G protein-coupled receptor ChenmR23, which has been detected in leukocyte populations such as macrophages, natural killer cells, and dendritic cells [17]. The interaction of chemerin with ChemR23 leads to migration of the cells to injured sites [18].

In the previous study, we tried to test whether hMSCs can alleviate liver fibrosis after injury and administered hMSCs via the systemic route in mouse model of liver injury using thioacetamide (TAA) [19]. We observed homing of several hMSCs to the injured liver, leading to reduction of liver fibrosis. In the current study, we aimed to identify the hMSC chemoattractant that is secreted from the injured tissue to improve the homing efficiency of hMSCs to the injured liver and then maximize the therapeutic effect of hMSCs. We analyzed the expression profile of chemokines from the injured liver and discovered chemerin as a practical tool to enhance the homing of hMSCs after systemic administration into target tissue.

## Results

### Screening chemoattractant of hE-MSC toward the injured liver

**Fig. 1:**
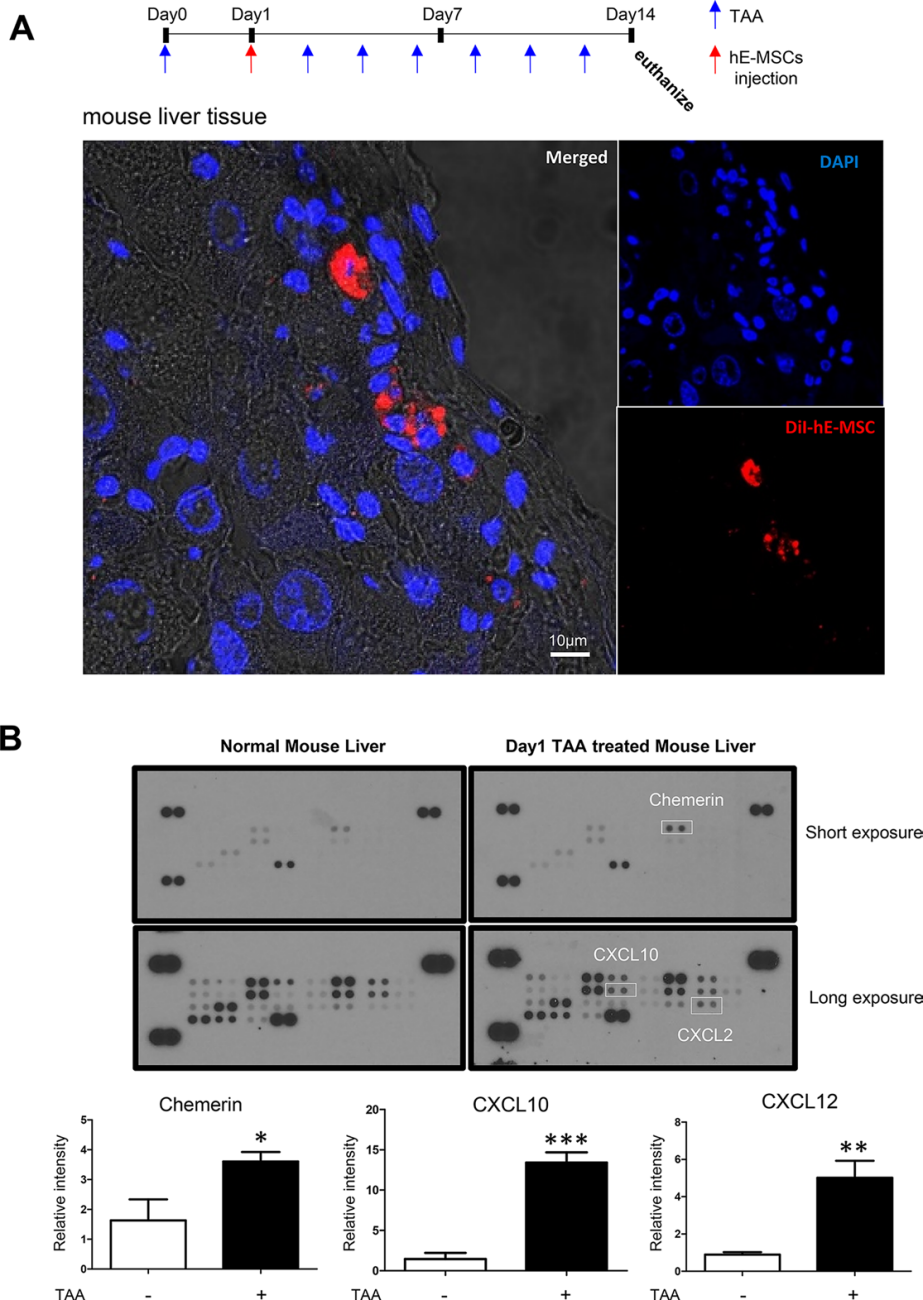

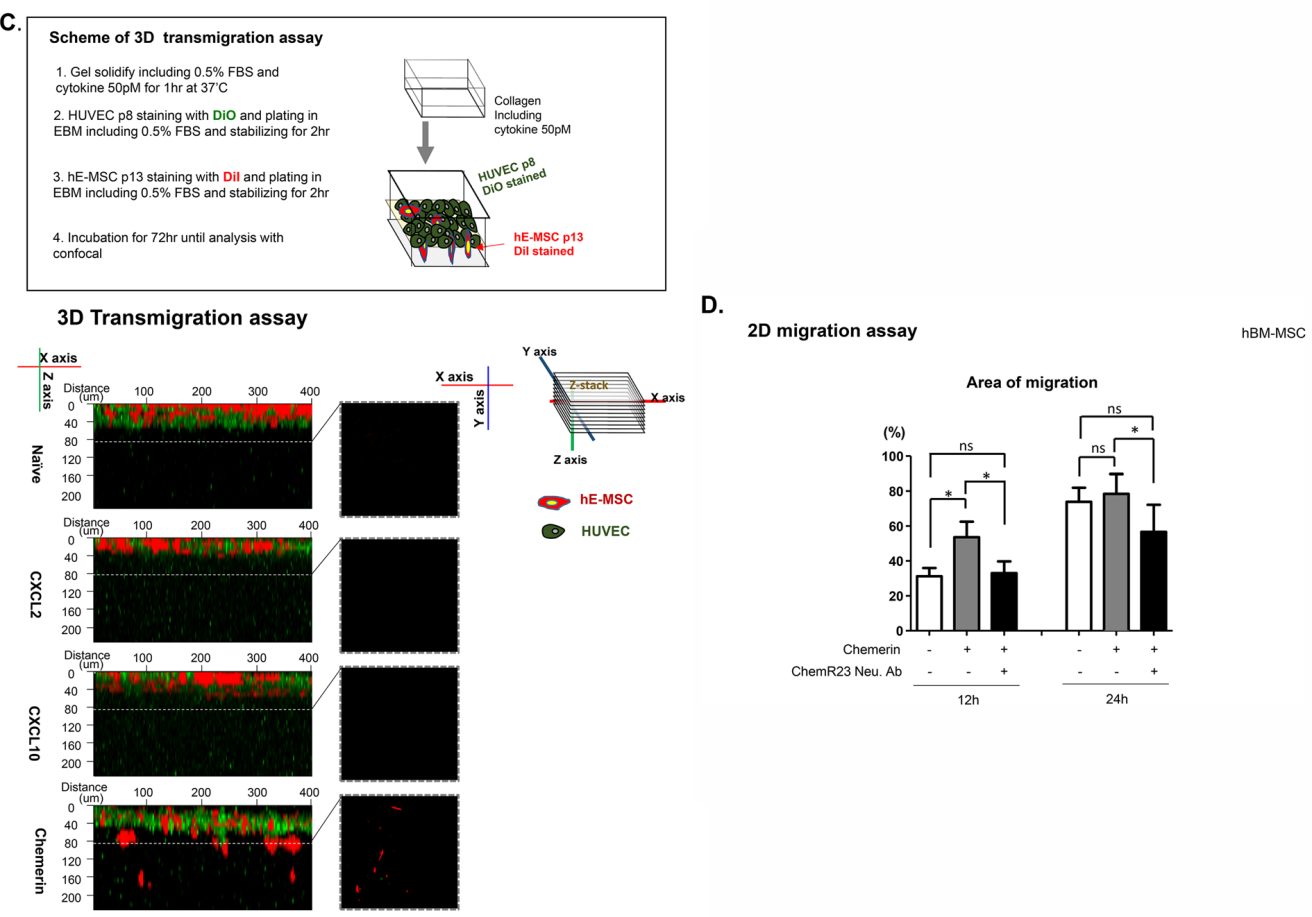
Screening of chemoattractant candidates for hMSCs. **A** Liver fibrosis model was generated by injecting TAA (0.2 mg/mL) every 2 days to the mouse. DiI-labeled hE-MSCs were systemically administered after the first TAA injection. DiI-labeled hE-MSCs were found in mouse liver treated with TAA. DiI hE-MSCs are red and the nuclei are blue. **B** Mouse chemokine array blots of normal and the injured liver at 1-day after TAA-treatment. The upper blot was exposed for 5 min and the bottom blot was exposed for 10 min. Chemokines are shown as duplicate dots. Reference dots are shown at the periphery; top right and left as well as bottom left. White squares indicate chemokines that were significantly increased in the injured mouse liver with TAA (0.2 mg/mL). The data shown were reproducible results from two independent experiments. Blots were quantified by Image J software. (C) 3D transmigration assay. Upper panel shows the scheme. The 3D transmigration system constructed using DiO-labeled HUVECs (green) as the endothelial layer on the surface of collagen gel below containing a given chemokine such as CXCL2, CXCL10, or chemerin (all at 50 pM). DiI-labeled hE-MSCs (red) were seeded on HUVECs and their migration into collagen gel was observed for 72 h by performing Z-stacking in a confocal microscope. Migration distances of hE-MSCs are indicated on a scale of 40 µm. The data shown are reproducible results from two independent experiments. **D** 2D random migration assay of hBM-MSC in response to chemerin or ChemR23 blocker (ChemR23 neutralizing antibody). Quantification of cell migration at 12 and 24 h was represented as the percentage of cell coverage in the initial cell-free zone. The white bar represents control hBM-MSCs, the gray bar indicates hBM-MSCs exposed to chemerin in media, and the black bar indicates hBM-MSCs that were treated with the ChemR23 neutralizing antibody in addition to chemerin

Previously, we have constructed a mouse liver fibrosis model using a well-established TAA-induced liver fibrosis protocol [19, 20]. hE-MSCs were injected according to the time table shown in Fig. 1 to confirm the therapeutic effect of hE-MSCs. To visualize the cells, we labeled hE-MSCs with DiI and systemically administered them one day after TAA treatment. We observed the DiI-labeled hE-MSCs that infiltrated to liver at day 14 after systemic administration (Fig. 1A). To screen potential chemoattractants in liver, the cytokine array was used on normal and TAA-treated liver tissue. Short exposure of the film showed that the chemerin levels in TAA-treated liver were higher than that in the normal liver. Longer exposure showed increase in the expression of CXCL2 and CXCL10 also (Fig. 1B).

To validate three candidates, a 3D transendothelial migration system was constructed using a DiO-labeled HUVEC layer on top of collagen which was supplemented with chemokines (CXCL2, CXCL10, and chemerin). We applied DiI-labeled hE-MSCs on HUVEC layer and observed for 72 h that hE-MSCs migrated through HUVEC toward chemerin-supplemented collagen matrix but not toward CXCL2- or CXCL10-supplemented collagen (Fig. 1C).

To determine whether chemerin can be used on other cell types, we checked the applicability of chemerin in hBM-MSCs. Chemerin attracted hBM-MSCs also in the 2D migration assay, which was blocked by the neutralizing antibody against chemerin (Fig. 1D).

### Chemerin induced migration-related gene expression in hBM-MSCs

**Fig. 2:**
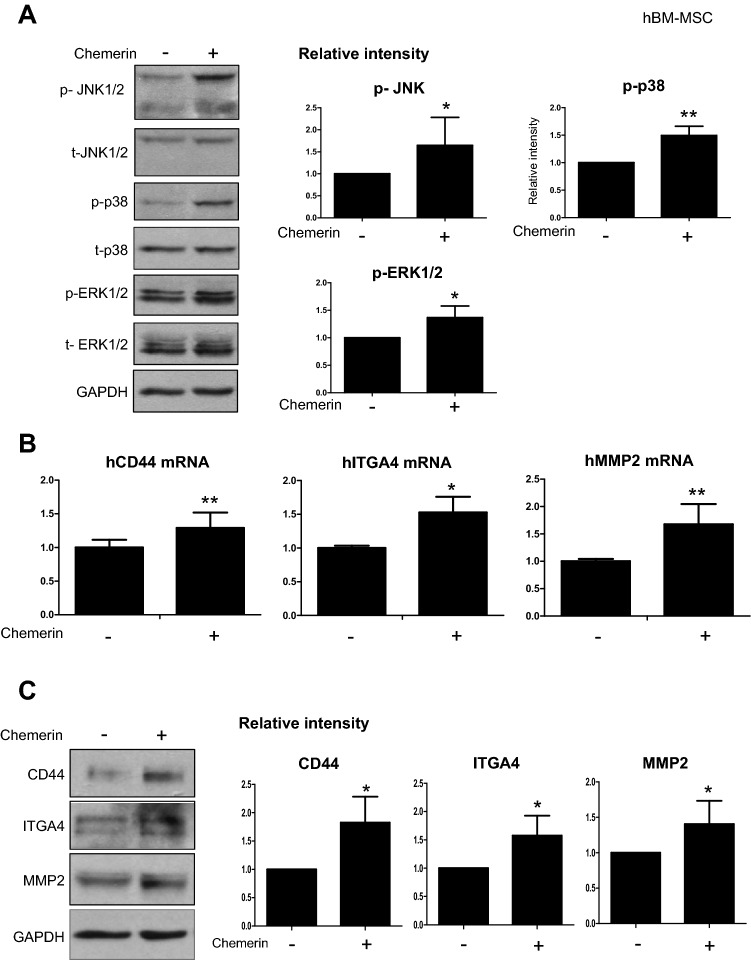

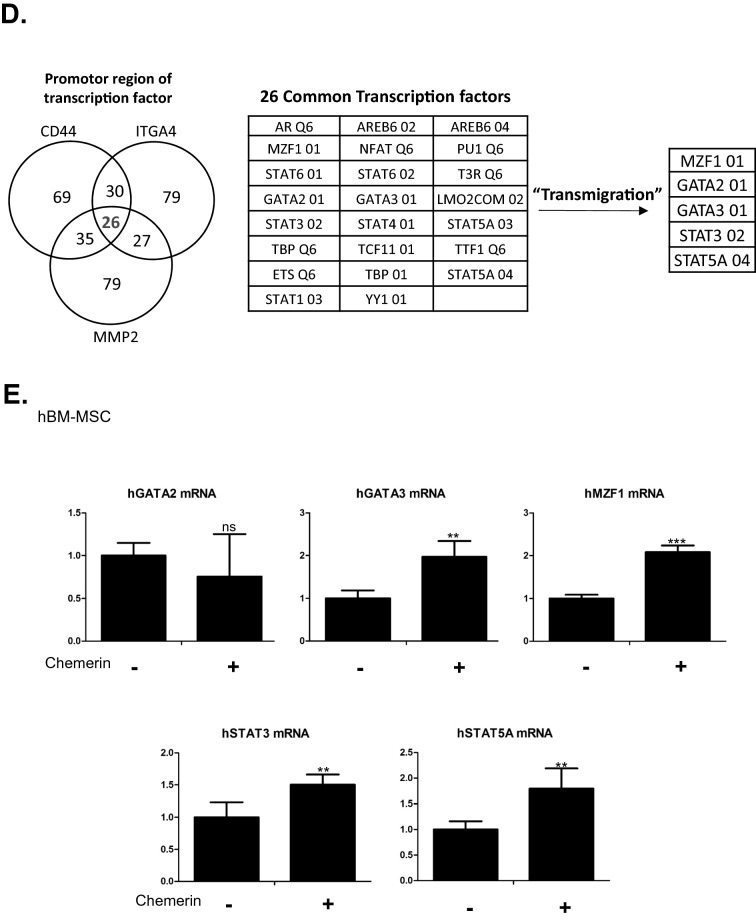
Chemerin induced phosphorylation of JNK1/2, ERK1/2, and p38 and increased the expression of migration-related genes in hBM-MSCs. **A** Western blot of phosphorylated JNK1/2, p38, and ERK1/2 in hBM-MSCs after chemerin treatment. Protein expression was quantified using the Image J software. Expression was normalized to that of GAPDH (N > 3). **B** RT-qPCR of CD44, ITGA4, and MMP-2 after chemerin treatment. mRNA levels were observed 24 h after the chemerin treatment of BM-MSCs. **C** Western blot analysis of CD44, ITGA4, and MMP-2 of hBM-MSCs after chemerin treatment. Protein expression was quantified using Image J and normalized to GAPDH level. **D** Venn diagram of common transcription factors, CD44, ITGA4, and MMP2. Transcription of each gene was found using GP miner and 26 genes were found to encode common transcription factors. Among these transcription factors, five were involved in transmigration. GP miner (http://gpminer.mbc.nctu.edu.tw/). **E** RT-qPCR of transcription factors in naïve and chemerin-treated hBM-MSCs. The data shown were reproducible results from three independent experiments

To determine the action mechanism of chemerin in hBM-MSCs, first we checked the phosphorylation status of JNK1/2, ERK1/2, and p38, which have been reported to be activated when chemerin interacts with ChemR23 [21]. Chemerin significantly increased the phosphorylation status of these proteins (Fig. 2A). Next, we assessed the expression of transmigration-related genes of MSCs, including *CD44, ITGA4*, and *MMP2*, and observed increase in mRNA and protein levels of these genes (Fig. 2B, C). To understand the regulatory mechanism, the common transcription factors that bind to the promoter regions of *CD44, ITGA4,* and *MMP2* were screened using the Gene Promoter Miner to detect common transcription factors under the key word ‘transmigration’. And then the screened factors were filtered again by searching for research papers on transmigration in NCBI (National Center for Biotechnology Information) (Fig. 2D) [22–26]. The common transcription factors that bind to the promoter regions of *CD44, ITGA4,* and *MMP2* and are involved in transmigration were *GATA3, MZF1, STAT3*, and *STAT5A* (Fig. 2D). The mRNA of these four factors increased by treatment with chemerin (Fig. 2E).

### Construction of chemerin vector to attract hMSC in vitro model

**Fig. 3:**
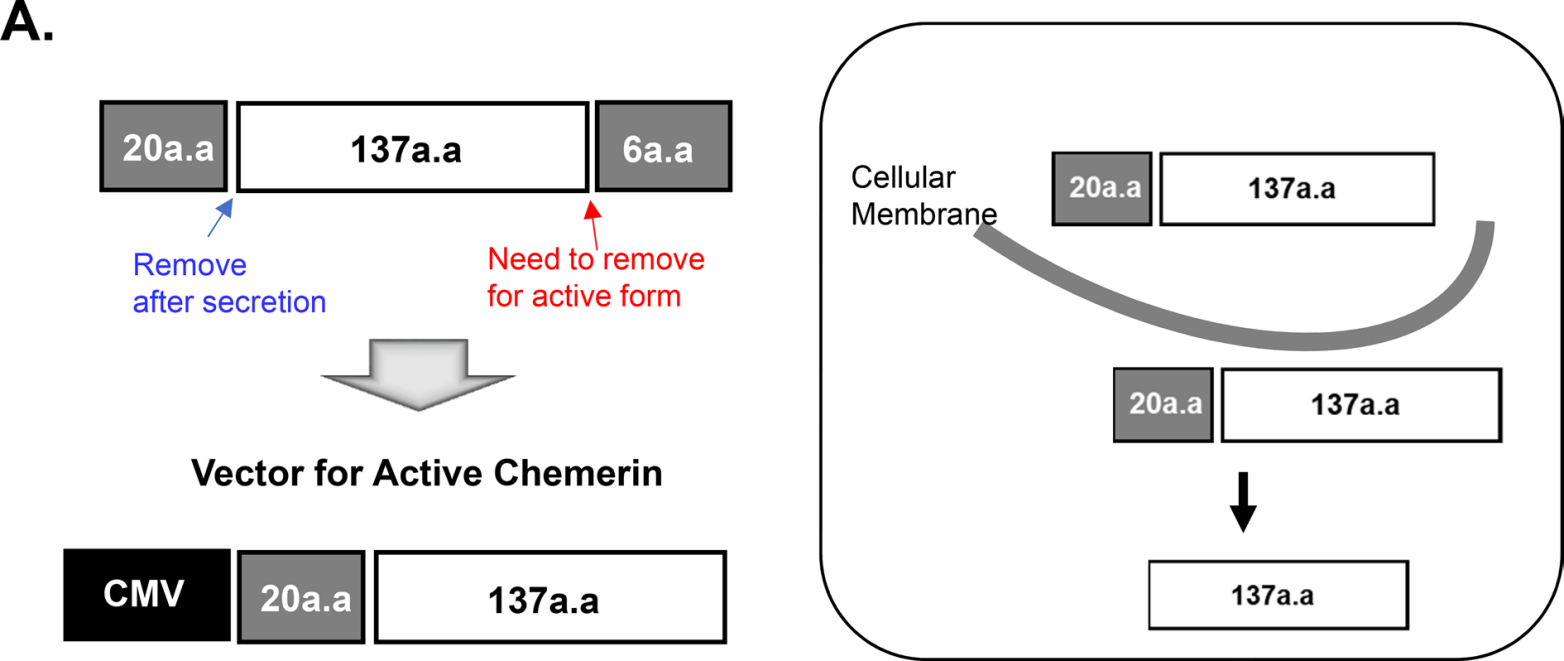

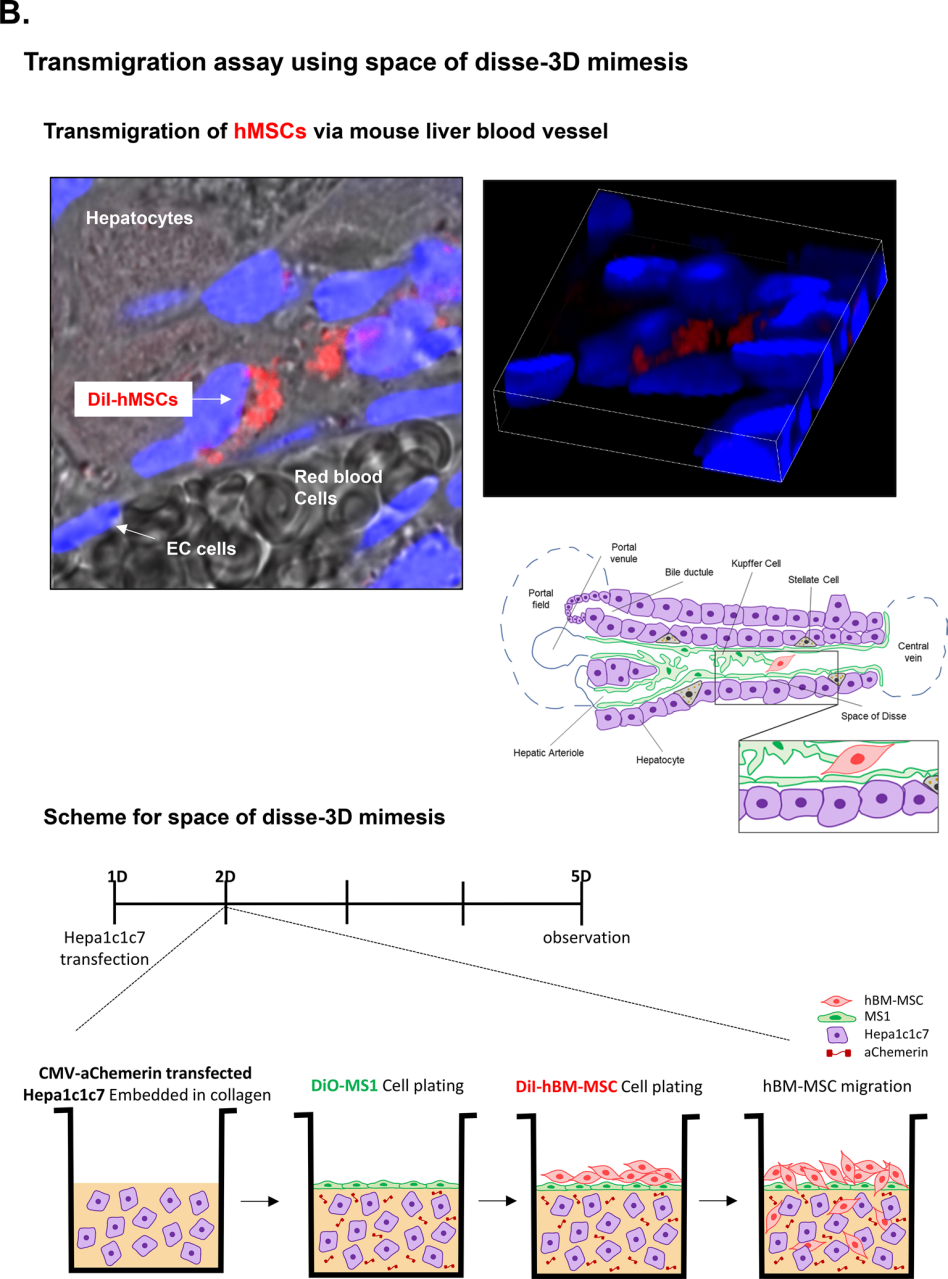

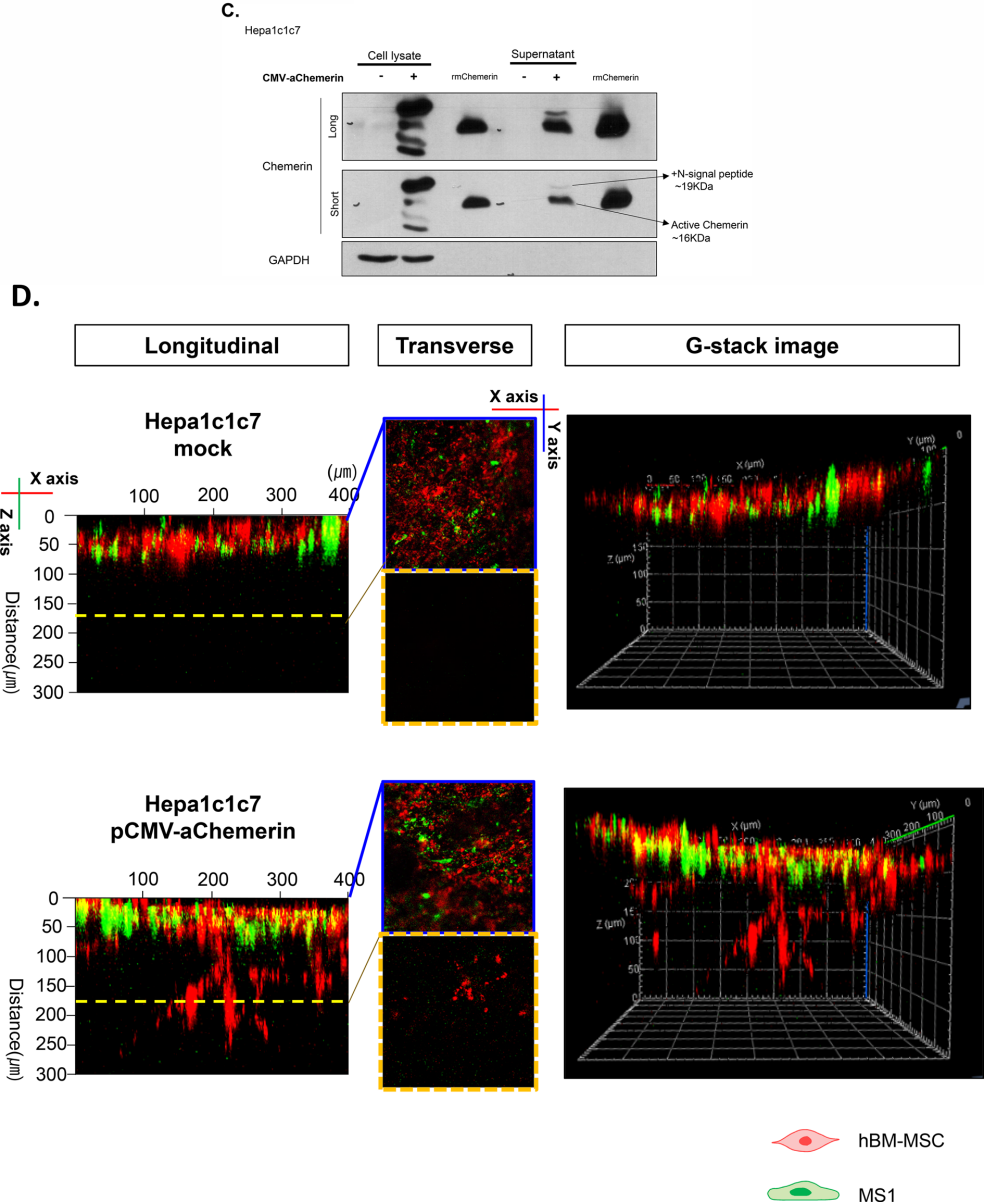
Construction of active chemerin vector. **A** Scheme showing the active chemerin vector. Twenty amino acids at N-terminus represent the signaling peptide that assists secretion of chemerin from hMSCs. The last six amino acids at C-terminus are enzymatically cleaved away leaving active chemerin. **B** Scheme for 3D mimesis of the space of Disse. The upper panel shows the possibility of transendothelial migration of hBM-MSCs in the space of Disse. To observe the possibility of ex vivo migration, a similar environment was constructed using Hepa-1c1c7 (hepatocytes), MS-1 (endothelial cells), and hBM-MSCs. **C** Evaluation of the vector in Hepa-1c1c7. Levels of the vector-encoded protein in the lysate and media of Hepa-1c1c7 cells transfected with the vector were detected using western blotting. The cell lysate shows different-sized chemerin and the supernatant shows inactive and active chemerin. Recombinant mouse chemerin (rmChemerin) was loaded as the positive control. **D** Transendothelial migration of hBM-MSCs. Penetration of hBM-MSCs through MS-1 cells into collagen matrix containing Hepa-1c1c7 cells transfected with active chemerin vector. MS-1 was labeled with DiO (green) and hBM-MSC was labeled with DiI (red). Migration in the vertical side of the collagen matrix was observed using the Z stack of the confocal microscope. The migration distances of hBM-MSCs were indicated in the scale of 50 µM. The data shown were reproducible results from two independent experiments

To use chemerin as a chemoattractant of hMSC, we designed a CMV-mouse chemerin vector (CMV-mChemerin) (Fig. 3A). Chemerin is activated after the cleavage of the last 6 or 7 amino acids at the C terminus depending on the enzyme. Hence, we designed this vector to encode an active form of chemerin that does not contain the last six amino acids, and thus can directly bind to ChemR23 without requiring any processing. Expression of this vector was detected and evaluated in 293 T cells. Active chemerin (~ 16 kDa) was detected in the cell culture supernatant and cell lysate (Additional file 1: Figure S1A). Interestingly, two bands were detected in the cell culture supernatant. The size of the upper band was 19 kDa reflecting inclusion of the signal peptide at the N-terminus, while the lower band was 16 kDa lacking the region. Using the culture supernatant of 293 T cells transfected with pCMV-aChemerin, we performed 2D migration of hBM-MSCs to confirm the function of the active chemerin construct. The random migration of hBM-MSCs was activated in the culture supernatant from cells transfected with the pCMV-aChemerin vector more than supernatant from un-transfected control cells (Additional file 1: Figure S1B).

Next, to validate the transendothelial migration, we developed a 3D transendothelial migration assay model, which mimics the transendothelial migration of MSCs in the space of Disse of the injured liver (Fig. 3B). We constructed this environment by (1) embedding in collagen the pCMV-aChemerin vector-transfected Hepa-1c1c7 cells (mouse hepatocyte) which represented the injured liver, (2) adding DiO-labeled MS-1 cells (mouse pancreatic islet endothelial cell) on top of this cell-collagen mix as an endothelial layer of the space of Disse, and finally (3) inoculating DiI-stained hBM-MSCs on the top of this structure. Active chemerin expression in Hepa-1c1c7 cells was confirmed using western blotting (Fig. 3C). We observed that hBM-MSCs penetrated the MS-1 layer and migrated effectively toward Hepa-1c1c7 cells transfected with CMV-aChemerin (Fig. 3D).

### The homing of systemically-administered hMSCs into liver by chemerin vector in liposome-vitamin A conjugate in vivo

**Fig. 4:**
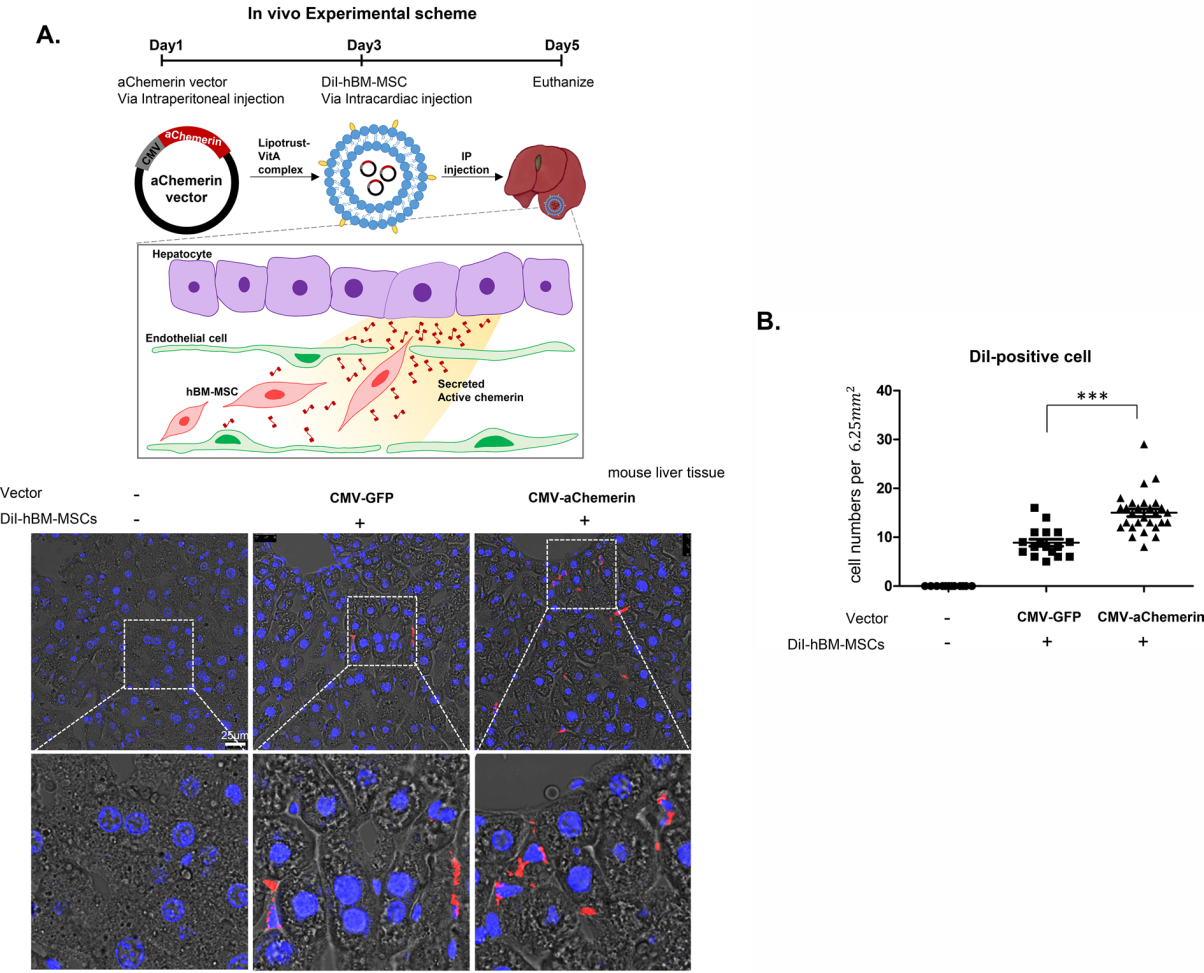
Applicability of the active chemerin vector in vivo. **A** Confocal microscopy images of DiI-labeled hBM-MSCs that homed to liver after systemic administration in mouse. Homing to liver was enhanced by pretreatment of pCMV-aChemerin vector in liposome-vitamin A conjugate. Red indicates DiI-labeled hBM-MSCs and blue indicates the nuclei. Confocal images were captured as a 6.25 mm^2^ square at random and the DiI-positive cells were counted manually (N > 20). **B** Results represent the mean ± standard deviation of two independent experiments

To validate the functionality of the chemerin vector to attract hMSCs in vivo, we intraperitoneally injected the vector into normal mouse. The vector was contained in liposome-vitaminA conjugate to target liver [19, 27]. Two days after vector injection, DiI-labeled hBM-MSCs were systemically administered via intra-cardiac injection. The livers were harvested 3 days after systemic introduction of DiI-hBM-MSCs to count the homing of these DiI-positive cells into liver. Homing of the systemically-administered hBM-MSCs to liver was significantly greater after introduction of pCMV-aChemerin in liposome-vitamin A conjugate than after pCMV-GFP control vector (Fig. 4 and Additional file 1: Figure S2).

## Discussion

### Limitation in clinical application of systemic transplantation of MSCs

The important issue in the clinical application of hMSCs that are most frequently used in real world practice is to achieve the best homing and engraftment efficacy to target tissue. Direct transplantation of hMSCs in the target region is the simplest method that cannot be applicable for most of the target organs. Instead, vascular delivery of hMSCs would be a plausible alternative one that has limitation such as poor homing or engraftment to the target organ. Transendothelial migration of MSCs that were introduced in the circulation may be the determinant of the homing efficacy of hMSCs to specific target tissue.

### Discovery of chemoattractant of hMSCs

In order to find out chemoattractant of the circulating hMSCs, we paid attention to the higher rate of homing of hMSCs to the injured liver than normal one after intravascular administration. We compared cytokine array between normal and the injured liver, and found that chemerin, CXCL2, and CXCL10 were the prominent three proteins that were higher in the injured liver than normal one. Among three factors only chemerin was the effective chemoattractant of hMSCs in the in vitro transendothelial migration assay.

In terms of CXCL10, it selectively recruits CXCR3-expressing T cells (CD4+, CD8+ and NK cells during inflammation [27]. When liver is inflamed, hepatocyte secretes CXCL10, thus recruiting CXCR3-expressing cells such as T cells, Kupffer cells, and hepatic stellate cells [28]. In terms of CXCL2, it is also produced by macrophages, monocytes, epithelial cells and hepatocytes during inflammation in liver. CXCL2 secreted form Kupffer cells attracts and activates neutrophils, leading to release of inflammatory mediators [29]. In terms of chemerin, it upregulates adhesion molecules on endothelial cells which enhances monocyte adhesion and development of atherosclerosis. Also, chemerin is involved in inflammation by activating p38 MAPK pathway in renal injury and inhibits CCL2 secretion in allergic asthma, leading to recruitment of inflammatory dendritic cells. Thus, when inflammation occurs, chemerin acts as an anti-inflammatory or pro-inflammatory mediator [30]. In the current study, chemerin is secreted from the damaged liver, acts as a chemoattractant of hMSC, leading to their homing to liver.

Transmigration of MSCs from the blood to a specific organ involves several steps. The first step is that the circulating MSC tether to endothelial cells via interaction between CD44 on MSCs and selectin on endothelial cells [28]. Next, VLA4 (heterodimer of ITGA4 and ITGB1) of MSCs interacts with VCAM1 of endothelial cells, resulting in the rolling and arrest of MSCs [28, 29]. Finally, MMP-2 secretion is required for the invasion of MSCs through the basement membrane of the endothelium and cleavage of the extracellular matrix for MSCs to arrive at the desired target [28, 29]. In this study, we observed that CD44, ITGA4, and MMP-2 in MSCs were induced by chemerin. We deciphered the upstream regulator mechanisms, such as, phosphorylation of JNK1/2, ERK1/2 and p38, and then induction of transcription factors MZF1, GATA3, STAT3, and STAT5A.

### Active chemerin as a practical tool to guide hMSCs into target organ

**Fig. 5:**
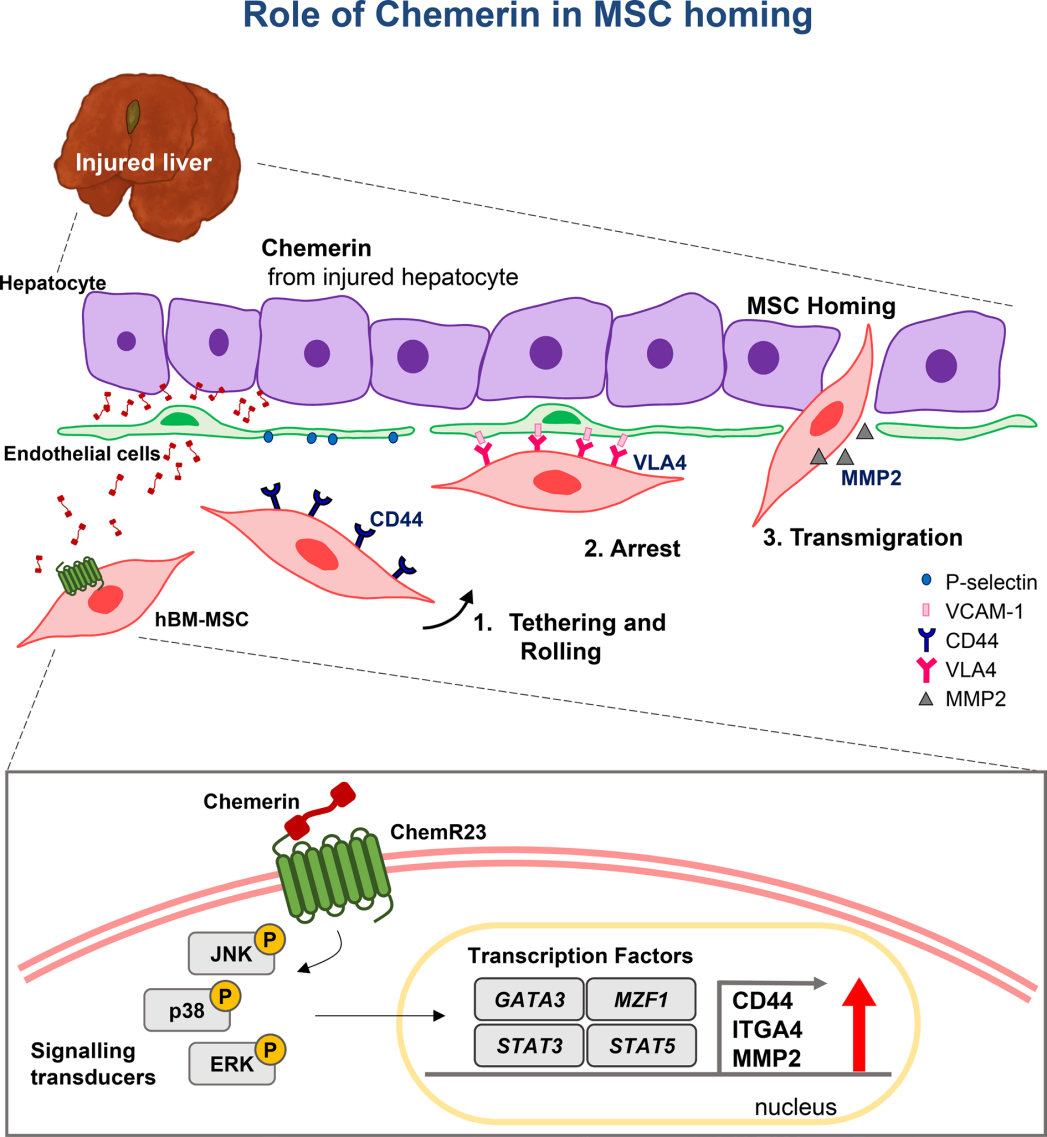
Schemes explaining the mechanism how chemerin chemo-attracts the circulating MSCs to the injured liver. Chemerin secreted from the injured liver interacts with ChemR23 on surface of MSCs, leading to activation of adhesion molecules that mediate steps of trans-endothelial migration of the circulating MSCs to the injured liver

Our results indicated that chemerin stimulated the expression of transmigration-related genes via these transcription factors (Fig. 5). We observed up-regulation of *MMP-2* by MSCs in response to chemerin, as well as *CD44* and *ITGA4*. Previous studies have shown that MMP-2 is required for transendothelial migration of MSCs [30]. Therefore, for practical application of chemerin, we constructed an active chemerin which does not require processing for activation. The signal peptide (20 amino acids) was retained for extracellular secretion. Proteins of various sizes were observed in the cell lysate, including chemerin with the signal peptide (~ 19 kDa) as well as the active chemerin of the predicted size (~ 16 kDa) in the culture supernatant. Secreted active chemerin attracted hMSCs through the endothelial barrier into target chamber ex vivo or into liver in vivo. The vector was delivered via the non-invasive route and targeted to the liver via liposome-vitamin A conjugate. However, further studies are required to develop the vector as a practical tool that can be delivered easily to other organs.

## Material and methods

### Cell culture

Human MSCs derived from embryonic stem cells (hE-MSCs) were obtained using a previously reported protocol [31]. hE-MSCs were cultured in EGM-2MV medium (Lonza, Switzerland, CC-3202). Bone marrow MSCs (hBM-MSCs) were purchased (Lonza; PT-2501) and cultured in mesenchymal stem cell growth medium 2 (Promocell, Germany; C-28009). Hepa-1c1c7 cells (ATCC, United Kingdom; CRL-2026) were cultured in 10% fetal bovine serum (FBS)-supplemented minimal essential medium (MEM) (Thermo Fisher Scientific, USA; 11095080). HUVECs (Lonza; CC-3202) were cultured in EGM 2MV (Lonza; CC-3202). MS-1 cells (ATCC; CRL-2279) were cultured in 5% FBS-supplemented Dulbecco’s modified Eagle’s medium (DMEM) (Thermo Fisher Scientific; 11995065). FBS was from Gibco (Thermo Fisher Scientific; 16000). All cells were incubated at 37 °C in the presence of 5% CO_2_.

### Chemokine array

The Proteome Profiler Mouse Chemokine Array kit (R&D system, United State, ARY020) was used according to the manufacturer’s protocol.

### Western blotting

Cells were harvested in radioimmunoprecipitation assay (RIPA) buffer (Thermo Fisher Scientific; 89900) and were agitated on ice for 15 min, after which the lysates were centrifuged at 13,000 rpm, 4 ℃, for 10 min. Then, the supernatants were collected and quantified using the bicinchoninic acid (BCA) protein assay kit (Thermo Fisher Scientific; 23225). The proteins were loaded on 10% Tris–glycine sodium dodecyl sulfate (SDS)-polyacrylamide gel and transferred to membranes. The membranes were blocked in 5% normal horse serum (NHS) (Sigma-Aldrich, United State, H1270-500ML) for 1 h, followed by addition of primary antibodies and overnight incubation. Following are the primary antibodies used: anti-mChemerin (1:1000; R&D System, USA; AF2325), anti-phopspho-JNK (1:1000, Cell Signaling Technology, USA; 4668), anti-JNK (1:1000, Cell Signaling Technology; 9252), anti-phospho-p38 (1:1000, Cell Signaling Technology; 4511), anti-p38 (1:1000, Cell Signaling Technology; 8690), anti-phospho-ERK1/2 (1:1000, Cell Signaling Technology; 4370), and anti-ERK1/2 (1:1000, Cell Signaling Technology; 4695). An anti-GAPDH (1:5000, Thermo Fisher Scientific; MA5-15738) antibody was used as an internal housekeeping control. The immunoblotted membranes were incubated with horse radish peroxidase (HRP)-conjugated secondary antibodies (1:2000, Jackson ImmunoResearch Laboratories).

### Quantitative polymerase chain reaction (qPCR)

RNA was isolated from cells using TRIzol (Invitrogen, United State, 15596026) per the manufacturer’s protocol and RNA concentration was quantified using a NanoDrop (Thermo Fisher Scientific; ND-ONE-W^4^). One microgram RNA was transcribed into cDNA using the RT master premix (oligo d(T) kit (Elpis Biotech, Korea; EBT-1512). All reactions were made using FastStart Universal SYBR Green master (Rox) mix (Merck Millipore, Germany; 4913850001). RT-qPCR was performed for 40 cycles of 95 ℃ for 15 s, 60 ℃ for 15 s, and 72 ℃ for 30 s using ABI Prism 7500 (Applied Biosystems, USA).

The sequences of the primers used are as follows:

hCD44 Forward: 5′CGAAGAAGGTGTGGGCAGAAG 3′

hCD44 Reverse: 5′CGACTCCTTGTTCACCAAATGC 3′

hITGA4 Forward: 5′TAGCCCTAATGGAGAACCTTGTG 3′

hITGA4 Reverse: 5′TCTATGCCCACAAGTCACGATG 3′

hMMP2 Forward: 5′GCCAAGTGGTCCGTGTGAAG 3′

hMMP2 Reverse: 5′CAAAGTTGTAGGTGGTGGAGCA 3′

hGATA2 Forward: 5′ GCCACAGCCACCCCTCTC 3′

hGATA2 Reverse: 5′ GGTTGTCGTCAGTCTTCGCTT 3′

hGATA3 Forward: 5′ CACCCCATCACCACCTACCC 3′

hGATA3 Reverse: 5′ CCTGCCTGTGCTGGACCG 3′

MZF1 Forward: 5′ CTTCTCCCCAGGGTTCGC3′

MZF1 Reverse: 5′ GCGGGAGGGTGATTGGAT 3′

hSTAT3 Forward: 5′CTAGAGACCCACTCCTTGCCAG 3′

hSTAT3 Reverse: 5′TTTACATTCTTGGGATTGTTGGT 3′

hSTAT5A Forward: 5′GTCCTGAAGACCCAGACCAAGT 3′

hSTAT5A Reverse: 5′CTCGTTGCGGGTGTTCTCAT 3′

### 2D migration assay

hBM-MSCs (1.0 × 10^4^) were plated in each of the ibidi culture-insert 2 wells (ibidi, USA; 81,176) and incubated overnight in desired media at 37 °C in the presence of 5% CO_2_. The next day, the insert 2 well was removed and the medium was replaced with 0.5% FBS. Next, 50 pM of mChemerin (R & D Systems; 2325-CM-025), mCXCL2 (PeproTech, USA; 205-15), and mCXCL10 (PeproTech; 250-16) were added to the cells. The cells were observed and images were captured after every 6 h. The images were quantified using Image J.

### 3D transmigration

Three hundred microliters of the collagen mixture from 3D collagen cell culture system (Merck Millipore, USA; ECM 675) were plated in eight wells (ibidi, United State 80826) and solidified in a 5% CO_2_ incubator for 1 h. After solidification of collagen, 5 × 10^4^ DiO (Thermo Fisher Scientific; V22886)-labeled HUVECs were plated. Once the HUVECs had attached, 5 × 10^4^ DiI (Thermo Fisher Scientific; C7001)-labeled hE-MSCs were plated. These cells were incubated for 3 days and observed using a confocal microscope (Leica Microsystem, South Korea; Leica STED CW).

### 3D mimesis model for space of Disse

Hepa-1c1c7 cells transfected with pCMV-Flag-tagged mouse chemerin were trypsinized and mixed with collagen from the 3D collagen cell culture system (Merk Millipore; ECM 675). Three hundred microliters of the collagen-cell mixture were plated in eight wells (ibidi, United State 80826) and the collagen was solidified in 5% CO_2_ incubator for 1 h, following which, 5 × 10^4^ DiO (Thermo Fisher Scientific; V22886)-labeled MS-1 cells were plated. Once the MS-1 cells had attached, 5 × 10^4^ DiI (Thermo Fisher Scientific, United State, C7001)-labeled hBM-MSCs were plated. These cells were incubated for 2 days and then observed using a confocal microscope (Leica Microsystem).

### Vector construction

Prochemerin has a signaling peptide at the N-terminus and is enzymatically cleaved at the C-terminal. For generating active chemerin, we constructed a pCMV-aChemerin vector that included the signal peptide at the N-terminus and lacked the last six amino acids at the C-terminus. pCMV-mGFP (Origene) was used as the backbone vector.

### Mouse liver fibrosis model and cell transplantation

All animal study protocols were approved by the Institutional Animal Care and Use Committee (IACUC) of the Seoul National University Hospital, Korea. The IRB number is H-1410-093-619. BALB/c-nude mice (male, 12–13 weeks old, 20–25 g) (Orient, South Korea) were used for all animal experiments. Mice were administered 200 mg/kg TAA (Sigma-Aldrich; 163678) via intraperitoneal injection thrice per week for 14 days. Animals in the negative control group were injected with 0.9% saline. Cells were transplanted via intracardiac injection 1 day after the first TAA injection (Fig. 1A). Prior to cell injection, the hE-MSCs were stained with 1 mg/mL CellTracker CM-DiI (Thermo Fisher Scientific; C7000) and incubated at 37 ℃ for 24 h. Fourteen days’ post-cell transplantation, liver tissue was harvested for histology.

### CMV-aChemerin vector injection

The CMV-aChemerin vector and emerald GFP vector (OriGene) were incubated with the lipotrust-vitamin A complex. Lipotrust (CSR-LEO-10-EX, Cosmobio): retinol (Sigma; R7632) were mixed in ratio of 240:120 (nmol). Then, 18.5 µg vector was added to 100 nmol of the retinol:lipotrust complex and incubated for 20 min at room temperature. After 20 min, the complex was filtered using a PES column (Sartorius, Germany; VS0221) at 1500×*g* for 5 min at 25 ℃. Then, this complex was eluted with phosphate buffered saline (PBS) to a total volume of 70 µL and was intraperitoneally injected into BALB/c-nude mouse that were anesthetized with tribromoethanol. Two days after vector injection, hBM-MSCs were injected via cardiac injection. For hBM-MSC injection, 1.0 × 10^6^ hBM-MSCs were collected and incubated with CM-DiI dye (1 mg/mL) (Thermo Fisher Scientific; C7000) for 5 min at 37 °C and then incubated at 4 ℃ for 10 min. After 15 min, the cells were centrifuged at 1200 rpm for 5 min and washed twice with PBS. Then, the cells were suspended in PBS and 1.0 × 10^5^ were injected into each mouse. Two days after hBM-MSC injection (5 days after vector injection), mouse liver were harvested and fixed in 4% paraformaldehyde for 3 and 7 days or frozen in liquid nitrogen for protein analysis.

### Quantification of DiI-positive cells in vivo

Tissue harvested from mouse were fixed in 4% paraformaldehyde for 3 days and exchanged in 15% and 30% sucrose gradually until the tissue sank. Then, the tissue block was embedded in optimal cutting temperature compound (OCT) (Sakura Finetek, United State, HSK-4583) below − 20 ℃. Next, these tissues were cryosectioned at 7 µM. The nuclei were stained with 4′,6-diamidino-2-phenylindole (DAPI) for 15 min in PBS and washed thrice with PBS. DiI-hBM-MSCs were observed in each group using a 555 nm laser of a Leica confocal microscope.

### Statistical analysis

Experiments and groups were performed in triplicates, and all data were calculated as mean ± SD. Group comparisons were performed using the T-test, and the number of asterisks on the top of each graph indicated statistical significance. *,**, and *** indicated that the *p*-value range was 0.01 to 0.05, 0.001 to 0.01, and 0.0001 to 0.001, respectively.

## Supplementary Information

**Additional file 1: Fig. S1**. (A) Evaluation of the vector in 293 T cells. Western blotting of cell lysate and supernatant of pCMV-mChemerin-transfected 293 T cells. (B) Evaluation of the vector by 2D migration assay. Under the supernatant of pCMV-aChemerin-transfected 293 T cells, 2D random migration of hBM-MSCs were analyzed at 6 and 12 h. **Fig. S2.** Evaluation of human cells engrafting in mouse liver tissue. Genomic DNA from mouse liver tissue was extracted and analyzed by PCR for presence of human genome using equal amount. Genomic DNA of 293 T cells was used as a positive control. Human genomic DNA-specific primers; forward: ATGCTGATGTCTGGGTAGGGTG, reverse: TGAGTCAGGAGCCAGCGTATG, were used.

## Data Availability

Not applicable.
