# Supplementary material for: Discovery of chemerin as the new chemoattractant of human mesenchymal stem cells
Source: Cell Biosci. 2021 Jul 1;11:120. doi: 10.1186/s13578-021-00631-3 (PMC8252297; doi:10.1186/s13578-021-00631-3)
Supplement: Supplementary file 1 — Additional file 1: Fig. S1. (A) Evaluation of the vector in 293 T cells. Western blotting of cell lysate and supernatant of pCMV-mChemerin-transfected 293 T cells. (B) Evaluation of the vector by 2D migration assay. Under the supernatant of pCMV-aChemerin-transfected 293 T cells, 2D random migration of hBM-MSCs were analyzed at 6 and 12 h. Fig. S2. Evaluation of human cells engrafting in mouse liver tissue. Genomic DNA from mouse liver tissue was extracted and analyzed by PCR for presence of human genome using equal amount. Genomic DNA of 293 T cells was used as a positive control. Human genomic DNA-specific primers; forward: ATGCTGATGTCTGGGTAGGGTG, reverse: TGAGTCAGGAGCCAGCGTATG, were used. [file 13578_2021_631_MOESM1_ESM.pptx]

## Slide 1
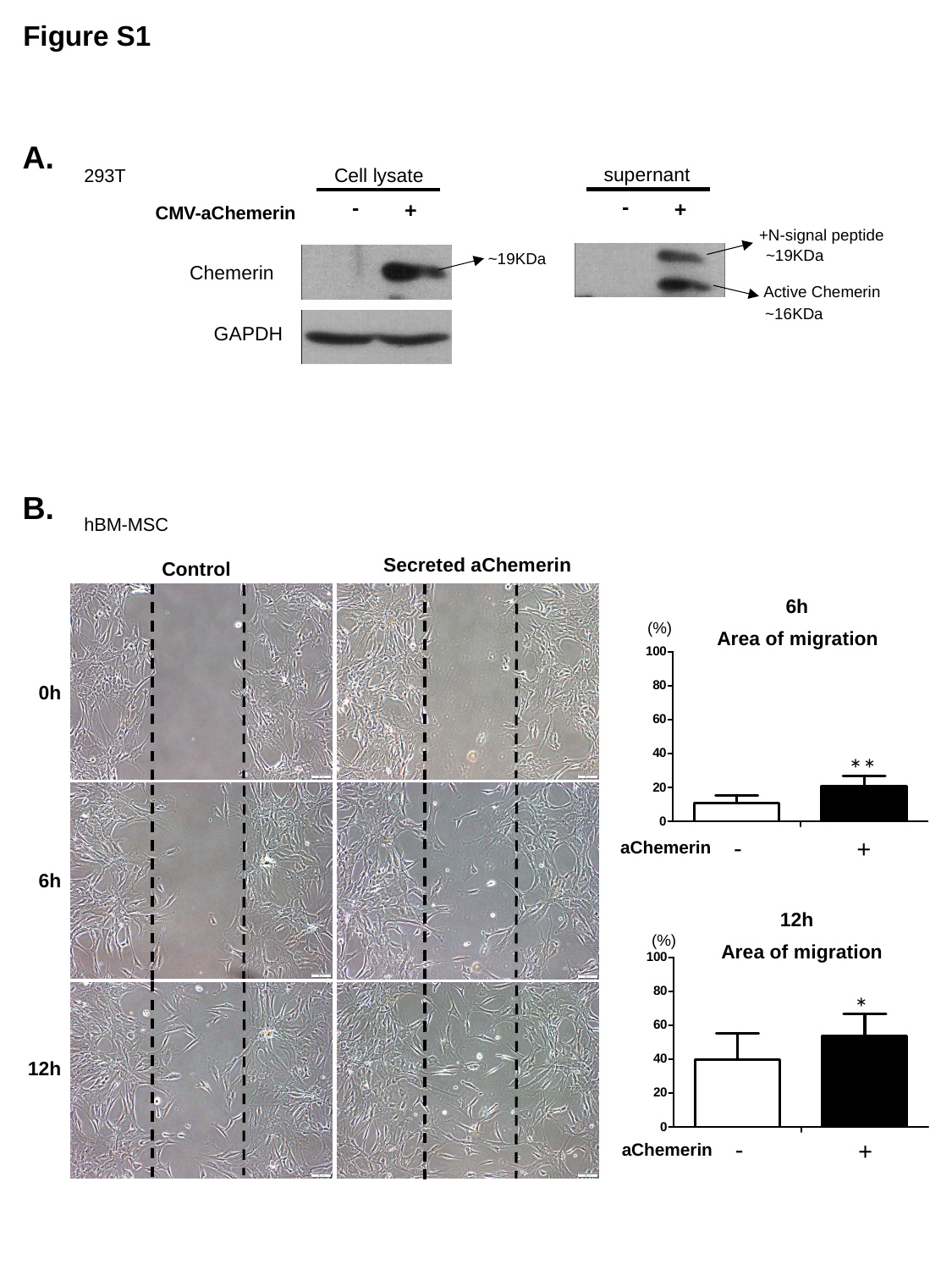

Figure S1
A.
supernant
Cell lysate
293T
-
-
+
+
CMV-aChemerin
+N-signal peptide
~19KDa
~19KDa
Chemerin
Active Chemerin
~16KDa
GAPDH
B.
hBM-MSC
Secreted aChemerin
Control
6h
(%)
Area of migration
0h
**
-
+
aChemerin
6h
12h
(%)
Area of migration
*
12h
-
+
aChemerin

## Slide 2
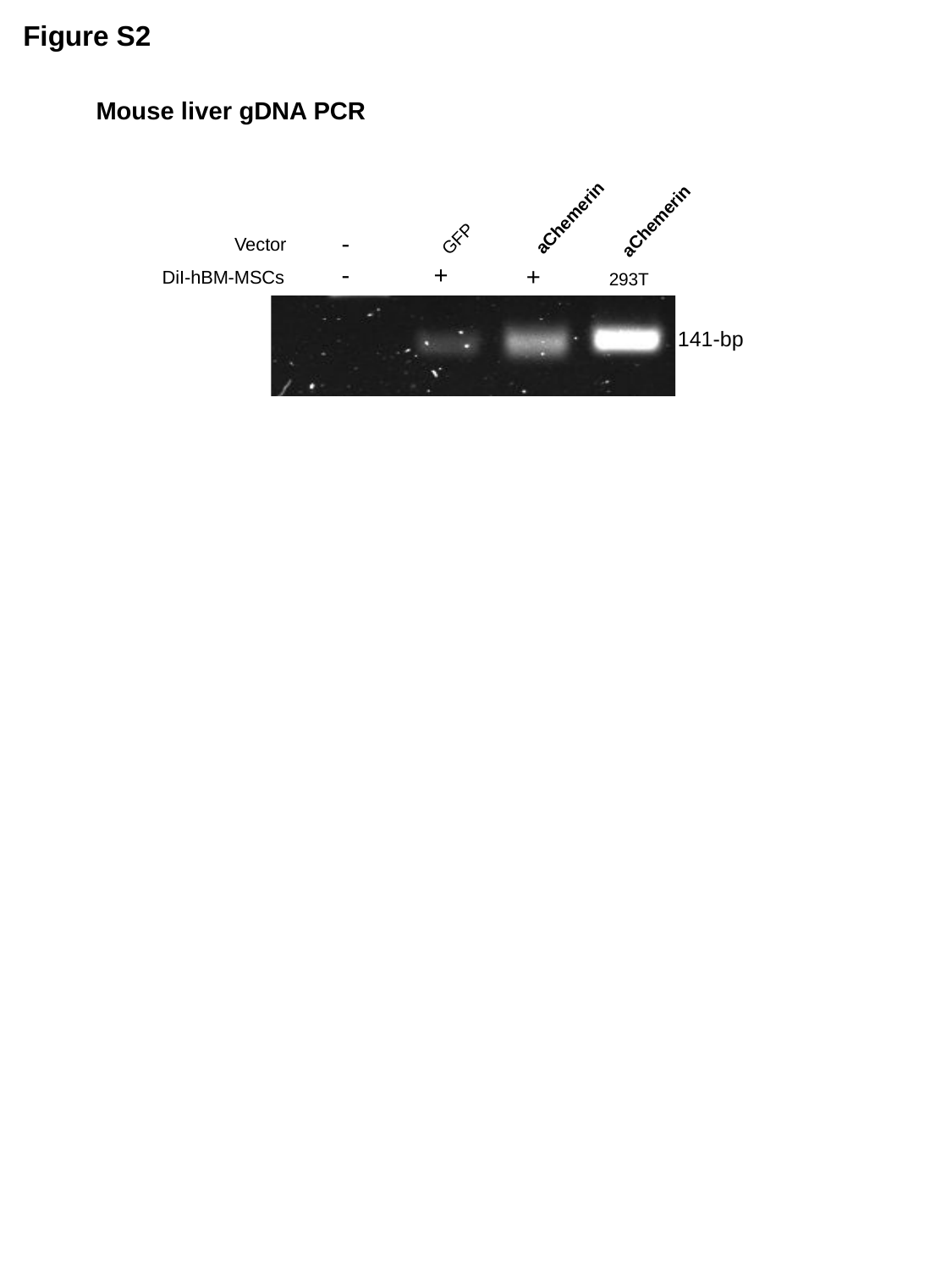

Figure S2
Mouse liver gDNA PCR
aChemerin
aChemerin
GFP
-
Vector
-
+
+
DiI-hBM-MSCs
293T
141-bp
